# First-in-human experience after integration of 4D ICE with intracardiac mapping system enabling magnetic navigation and image presentation during catheter ablation of cardiac arrhythmias

**DOI:** 10.1007/s10840-026-02380-0

**Published:** 2026-06-19

**Authors:** Ante Anic, Raphael Rosso, Toni Breskovic, Ivan Sikiric, Marija Dagelic, Marco Schiavone, Gaetano Fassini, Claudio Tondo

**Affiliations:** 1 Department of Cardiovascular Diseases, Division of Arrhythmias, University Hospital of Split, Šoltanska 1 (Lokalitet Križine), Split, 21000 Croatia; 2 Tel Aviv Sourasky Medical Center and Gray Faculty of Medical & Health Sciences, Tel Aviv University, Tel Aviv, Israel; 3 Department of Clinical Electrophysiology & Cardiac Pacing, Centro Cardiologico Monzino IRCCS, Milan, Italy; 4 Department of Biomedical, Surgical and Dental Sciences, University of Milan, Milan, Italy

Two-dimensional (2D) intracardiac echocardiography (ICE) is widely used in ablation procedures as an alternative to transesophageal echocardiography (TEE) [1] and has recently been recommended for atrial thrombus assessment [2]. However, 2D ICE does not allow real-time multiplanar visualization of anatomical structures, and TEE requires a separate operator and general anesthesia, and is contraindicated in patients with esophageal injury risk [3]. The NUVISION Ultrasound Catheter (Biosense Webster, Inc., Johnson & Johnson MedTech), which incorporates real-time multiplanar reconstruction of anatomical structures to address the limitations of 2D ICE and TEE, has shown acute success, minimal safety events, and an optimized workflow for left atrial appendage occlusion [4, 5]. Use of this catheter for atrial fibrillation (AF) ablation procedures requires electroanatomical mapping (EAM) integration with ICE imaging. Thus, the NUVISION NAV Ultrasound Catheter (Biosense Webster, Inc., Johnson & Johnson MedTech) was designed to interface with the CARTO 3 System (Biosense Webster, Inc., Johnson & Johnson MedTech), enabling EAM imaging.

This first-in-human, prospective, single-arm, open-label, multicenter study evaluated safety and performance of the NUVISION NAV catheter, a single-use, diagnostic ultrasound imaging catheter designed for ICE use, in participants undergoing image-guided cardiac ablation procedures. European regulatory bodies and ethics committees approved the study; all participants provided written informed consent. The catheter’s distal end has an ultrasound transducer with a 2D acoustic element array on an application-specific integrated circuit, enabling real-time 2D, 3D, and 4D imaging. It is coupled with a 3D magnetic sensor providing location information to the compatible EAM system.

Treatment was performed per standard of care. Investigational catheter use was mandatory for thrombus detection, volume measurement, transseptal puncture guidance, and ultrasound navigation. For AF ablation, CARTOSOUND-created geometry was merged with preprocedure computed tomography angiography. The primary safety endpoint was investigational catheter–related serious adverse events (SAEs) within 7 days of index procedure. The primary performance endpoint was completion of procedure-required imaging without using a nonstudy ultrasound device. Secondary endpoints included physicians’ assessments of the investigational catheter and non–device-related SAEs, and device-related nonserious adverse events (AEs) within 7 days of index procedure. All data were summarized descriptively with no formal hypothesis testing.

Thirty participants were enrolled from September 2023 to February 2024 at 2 European sites. Most (80%) participants had atrial arrhythmia (atrial tachycardia, *n* = 7; persistent AF, *n* = 5; paroxysmal AF, *n* = 12), and 20% had ventricular arrhythmia (ventricular tachycardia, *n* = 2; premature ventricular complex, *n* = 4). The mean ± SD age was 64.1 ± 13.4 years, and 53.3% of participants were male. The most common comorbidity was hypertension (40.0%). The mean ± SD baseline left ventricular ejection fraction was 58.7% ± 9.35%, and 46.7% of participants had prior ablation history.

The mean ± SD procedure and fluoroscopy times were 97.0 ± 35.2 and 10.1 ± 4.5 min, respectively. The mean ± SD left atrial geometry reconstruction time was 543.1 ± 324.8 s. Only 1 device deficiency (lack of connection with mapping system before sheath insertion) was recorded for the study catheter and was not associated with any AEs. The investigational catheter was used to guide transseptal punctures and assess a broad range of anatomical structures (Fig. 1). Preablation thrombus detection and imaging required for the study procedure were completed in all participants without using noninvestigational ultrasound catheters.

No participant experienced an SAE or AE related to the investigational catheter. Two SAEs were reported: vascular access site pseudoaneurysm (procedure related) and complete atrioventricular conduction block (not procedure related). There were 2 nonserious AEs: pericardial effusion (procedure related) and delayed wound healing (not procedure related).

Physicians were very satisfied with the investigational catheter deployment, maneuverability, navigational features, and EAM image quality. Most survey questions assessing catheter performance were scored as 6 or 7 (scale from 1 [dissatisfied] to 7 [most satisfied]), with a mean score of ≥ 6.0. Most physicians (87%) were willing to recommend the device to their peers.

This was a first-in-human, single-arm study in a limited population; a larger, controlled trial would be needed to confirm these findings. Nevertheless, the current study demonstrated the safety and performance of the NUVISION NAV catheter in various arrhythmia procedures. Optimal acute device safety and performance profiles were observed for intracardiac procedure–required imaging. Additionally, physician operators reported high satisfaction with the catheter’s performance.

**Fig. 1:**
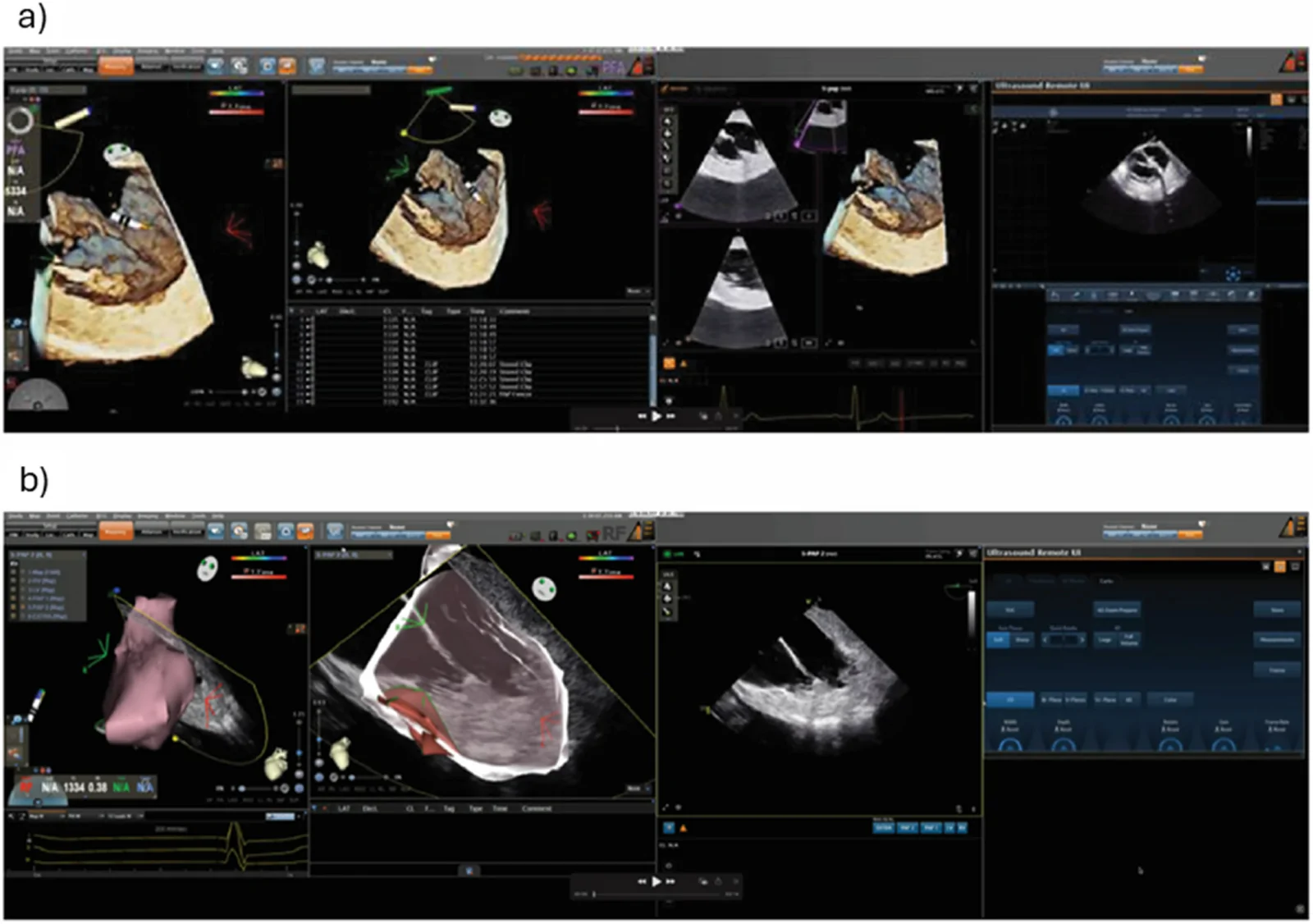
Examples of visualization using 4D ICE integrated with the CARTO 3 EAM System during the ablation procedure. Visualization directly onto a 4D ICE Freeze Bank (Biosense Webster, Inc., Johnson & Johnson MedTech) image during premature ventricular complex ablation originating in the papillary muscle (**a**) and CARTOSOUND 4D Freeze Bank (Biosense Webster, Inc., Johnson & Johnson MedTech) rendering and cropping the ultrasonic volume (**b**). 4D, 4-dimensional; EAM, electroanatomical mapping; ICE, intracardiac echocardiography. Images are courtesy of Biosense Webster, Inc., part of Johnson & Johnson MedTech. © All rights reserved

## Data Availability

Johnson & Johnson MedTech has an agreement with the Yale Open Data Access (YODA) Project to serve as the independent review panel for the evaluation of requests for clinical study reports and patient-level data from investigators and physicians for scientific research that will advance medical knowledge and public health. Requests for access to the study data can be submitted through the YODA Project site at http://yoda.yale.edu.
